# Comparisons between anosmia and ageusia with neurological symptoms and cardiorespiratory fitness among post-COVID-19 individuals: a cross-sectional study

**DOI:** 10.1007/s13760-026-03189-4

**Published:** 2026-09-11

**Authors:** Luciana Lozza de Moraes Marchiori, Glória de Moraes Marchiori, Fabiano Mendes de Oliveira, Victor Augusto Santos Perli, Maurício Medeiros Lemos, Braulio Henrique Magnani Branco

**Affiliations:** 1 Cesumar University (Unicesumar), Maringá, Paraná Brazil; 2 Interdisciplinary Laboratory for Health Promotion Intervention (LIIPS), Avenida Guedner, 1610 – Jardim Aclimação, Maringá, 87050-900 Paraná Brazil; 3 Cesumar Institute of Science, Technology and Innovation (ICETI), Maringá, Paraná Brazil

**Keywords:** Neurologic manifestations, Anosmia, Ageusia, COVID-19, Cardiorespiratory fitness

## Abstract

**Background:**

Anosmia and ageusia are among the most frequent symptoms of post-COVID-19 syndrome, yet their association with neurological symptoms and cardiorespiratory fitness remains unclear.

**Objective:**

To compare the presence of anosmia and ageusia with neurological symptoms and cardiorespiratory fitness in post-COVID-19 individuals.

**Methods:**

This cross-sectional study included 201 post-COVID-19 individuals who completed a standardized questionnaire assessing anosmia, ageusia, and neurological symptoms. Cardiorespiratory fitness was assessed using the Bruce treadmill test with direct measurement of peak oxygen uptake (VO₂peak). Missing data were handled by multiple imputation. Odds ratios (ORs) with 95% confidence intervals (CIs) were estimated using binary logistic regression, adjusted for age, sex, acute infection severity, medical history, body fat percentage, and skeletal muscle mass.

**Results:**

After adjustment, anosmia was independently associated with dizziness (OR = 2.22; 95% CI 1.15–4.31) and aural fullness (OR = 4.54; 95% CI 1.16–17.80), and ageusia with memory deficit (OR = 2.91; 95% CI 1.47–5.75) and aural fullness (OR = 4.08; 95% CI 1.02–16.30). No significant associations were found with tinnitus or hearing loss. VO₂peak did not differ significantly between participants with and without anosmia (mean difference 1.13 mL/kg/min; Cohen’s d = 0.14; 95% CI − 0.14 to 0.42; *p =* 0.325) or ageusia (mean difference 1.36 mL/kg/min; Cohen’s d = 0.17; 95% CI − 0.12 to 0.45; *p =* 0.244).

**Conclusions:**

Anosmia and ageusia are associated with neurological symptoms but not with cardiorespiratory fitness in post-COVID-19 individuals, suggesting partially independent phenotypes. These symptoms should be considered in therapeutic assessment and management.

## Introduction

Anosmia and ageusia were highly prevalent symptoms among individuals during or after COVID-19 [1]. However, the association between these conditions, cardiorespiratory fitness, and symptoms such as memory impairment, concentration difficulties, dizziness, and headache remains unclear [2, 3]. Cerebrovascular, neuropathic, and autonomic features of post-acute COVID-19 have been described, including cerebrovascular dysregulation with persistent cerebral arteriolar vasoconstriction, small-fiber neuropathy and related dysautonomia, respiratory dysregulation, and chronic inflammation. These findings suggest a relationship between various neurological manifestations and post-COVID-19 symptoms associated with inflammatory conditions, such as ageusia and anosmia, as well as metabolic and circulatory alterations in individuals after COVID-19 [4–6].

Furthermore, a growing number of studies suggest a relationship between olfactory and gustatory symptoms and post-COVID-19 complaints. Nevertheless, few studies have investigated neurological symptoms such as memory impairment, dizziness, and headache in association with ageusia and anosmia, as well as the relationship between cardiorespiratory fitness and these symptoms [4–8]. The study by Jung et al., involving 440 post-COVID-19 patients, suggests that these symptoms are related to other clinical factors, including fatigue, depression, anxiety, hyposmia, and hypogeusia [9].

Long COVID, which is characterized by a wide range of symptoms persisting after COVID-19, may be better understood through studies that examine symptom clustering after infection. In this context, a cohort study prospectively evaluated individuals with a previous history of severe COVID-19 who developed Long COVID over a two-year follow-up period. The study included symptom-cluster analysis using multiple correspondence analysis and hierarchical clustering in 199 patients with Long COVID. Three symptom clusters were identified: Cluster 1, characterized by fatigue, myalgia/arthralgia, a low prevalence of symptoms, and the absence of specific symptoms; Cluster 2, defined by a high prevalence of fatigue, myalgia/arthralgia, and cardiorespiratory symptoms, including palpitations, shortness of breath, cough, and chest pain; and Cluster 3, characterized by a high prevalence of ageusia, anosmia, fatigue, and cardiorespiratory symptoms [10].

Post-COVID syndrome is clinically heterogeneous, and not all patients present the same symptom burden, disability level, or healthcare needs. Population stratification according to disability level, symptom burden, and healthcare needs is therefore essential to optimize rehabilitation planning and resource allocation [11]. Multidimensional approaches that integrate demographic, clinical, and functional profiles may help identify subgroups of post-COVID patients who require differentiated care pathways and targeted rehabilitation strategies [11]. Based on this context, the present study aimed to compare the presence of anosmia and ageusia with neurological symptoms, including memory impairment, headache, and dizziness, as well as with cardiorespiratory fitness, among individuals after COVID-19.

## Methods

### Study design, setting, and population

This cross-sectional study is part of a broader investigation entitled the “Post-COVID-19 Project” and was approved by the institution’s Human Research Ethics Committee under Opinion No. 4.546.726. The study is reported in accordance with the The Strengthening the Reporting of Observational Studies in Epidemiology (STROBE) guidelines for observational studies [12]. All participants were informed about the study objectives, the guarantee of anonymity, and the assurance that their data would not be used for purposes other than those established in the research protocol. Prior to answering the study questions, all participants signed the informed consent form.

The study was conducted in a single center: the Interdisciplinary Laboratory for Health Promotion Intervention (LIIPS), Unicesumar, Maringá, Paraná, Brazil. All evaluations were performed in an outpatient, post-discharge research setting. Participants were identified through referrals from the municipal hospital of Maringá following hospital discharge [13]. Patients discharged after treatment for COVID-19 were referred to the LIIPS research laboratory; all eligible individuals referred during the recruitment period were consecutively invited to participate, and eligibility was confirmed by trained evaluators against the inclusion and exclusion criteria during the first laboratory visit. A total of 201 participants met the eligibility criteria and were included in the final analysis.

The inclusion criteria were as follows: being between 19 and 65 years of age; having a confirmed diagnosis of COVID-19 through a qualitative molecular test (RT-PCR), supported by presentation of the test result and/or hospital discharge documentation after treatment for COVID-19; having contracted COVID-19 between March 1 and July 1, 2021; having received the first dose of a COVID-19 vaccine; and presenting medical clearance to undergo cardiorespiratory fitness testing. The exclusion criteria comprised debilitating neurological diseases or mobility limitations requiring the use of a cane or wheelchair [10, 13]. Only neurological conditions meeting this severity threshold were excluded; participants with mild or non-debilitating neurological conditions were not excluded from the study.

## Data collection

Data collection was conducted by trained evaluators from the multidisciplinary team of the LIIPS, under the guidance of physicians, physical therapists, speech-language pathologists, nutritionists, and physical education professionals. During their first visit to the research laboratory, participants underwent a clinical assessment and completed a standardized questionnaire comprising 90 open- and closed-ended items. The questionnaire collected information on demographic characteristics, clinical history, pre-existing conditions (self-reported arterial hypertension, diabetes mellitus, and obesity), smoking status, need for hospitalization, duration and type of hospitalization, vaccination status, and the time elapsed from acute COVID-19 infection to the assessment. The questionnaire also recorded symptoms such as anosmia, ageusia, memory impairment, concentration difficulties, dizziness, and headache during and/or after COVID-19, as well as the duration of symptoms after hospital discharge.

Anosmia, ageusia, and the neurological symptoms investigated were self-reported by the participants; no objective olfactory or gustatory testing was performed. The questionnaire items used a dichotomous (yes/no) self-report format, and participants were asked whether each symptom was present during the acute phase of COVID-19, after hospital discharge, or both. Baseline physical activity level was not systematically assessed in this cohort; this is acknowledged as a limitation in the Discussion. Subsequently, body composition and anthropometric measurements were assessed [10, 13]. The Bruce Treadmill Test was then performed to measure oxygen consumption using a metabolic gas analyzer (VO2000; MedGraphics, Saint Paul, MN, USA), and participants’ peak oxygen uptake (VO₂peak) was used as the analytical variable [10, 13]. Time from acute COVID-19 infection to assessment, acute infection severity, hospitalization and ICU admission rates, and vaccination status are reported in Table 1.

**Table 1:** Sample characteristics of the present study

Age	47.7 ± 12.8
Gender	
Male	105 (52.2)
Female	96 (47.8)
Medical history	
SAS	64 (31.8)
Diabetes Mellitus	32 (15.9)
Dyslipidemia	17 (18.4)
Smoking^⸶^	40 (19.9)
Anthropometry and body composition	
BMI (kg/m²)	31.0 ± 5.7
SMM (kg)	29.4 ± 7.5
FM (kg)	32.2 ± 12.3
BF (%)	37.6 ± 10.1
Acute disease severity	
NH	65 (32.3)
Ward	66 (32.8)
ICU	70 (34.8)
Infection-evaluation time (d)	214.5 ± 116.4
Oxygen therapy	117 (58.2)
Mechanical ventilation	79 (39.3)

Categorical data are presented as absolute and relative frequencies (%). Numerical data are presented as mean and standard deviation (±). BF = body fat percentage; BMI = body mass index; FM = fat mass; ICU = intensive care unit; NH = non hospitalized; SAS = systemic arterial hypertension; SMM = skeletal muscle mass; ⸶ = currently or former smoker; per inclusion criteria (all participants received at least the first dose of a COVID-19 vaccine)

### Statistical analysis

Data distribution was assessed by visual inspection of distribution plots, together with skewness and kurtosis tests, with established tolerance limits of − 2 to 2. After checking for normality, continuous variables were described as means and standard deviations, and categorical variables as absolute and relative frequencies. Missing data were assessed before the analyses. Time from acute COVID-19 infection to assessment was missing for 15 participants (7.46%), body composition variables (BMI, fat mass, body fat percentage, and skeletal muscle mass) for 11 (5.47%), and VO₂peak for 8 (3.98%). After checking for registration errors, multiple imputation by chained equations (MICE) was used to handle missing data, and estimates were pooled across the imputed datasets according to Rubin’s rules. Crude and adjusted odds ratios (ORs) with their respective 95% confidence intervals (CIs) were estimated using binary logistic regression to analyze the associations between the presence of anosmia and ageusia and post-COVID neurological symptoms. Adjusted models included age, sex, acute infection severity (no hospital admission, hospital ward, intensive care unit), medical history (arterial hypertension, diabetes mellitus, dyslipidemia, and smoking), body fat percentage, and skeletal muscle mass as covariates. VO₂peak was compared between participants with and without anosmia and ageusia using independent-samples t-tests. Differences across symptom-duration categories were assessed using one-way analysis of variance (ANOVA); when a significant main effect was found, pairwise post-hoc comparisons with Bonferroni correction were performed, and Cohen’s d effect sizes with their 95% CIs were calculated. Associations between the presence of anosmia and ageusia and the remaining variables were analyzed using independent-samples t-tests for continuous variables and Fisher’s exact test for categorical variables. Statistical significance was set at *p <* 0.05 (two-sided). Analyses were performed using Jamovi (version 2.5.3.0) and RStudio (version 2023.09.1 + 494).

## Results

The characteristics of the study population are presented in Table 1. The mean age was 47.7 ± 12.8 years, and 105 (52.2%) participants were male. The prevalences of arterial hypertension, diabetes mellitus, and dyslipidemia were 31.8%, 15.9%, and 18.4%, respectively, and 40 (19.9%) participants were current or former smokers. Acute COVID-19 severity was high: 65 participants (32.3%) required no hospital admission, 66 (32.8%) were admitted to a hospital ward, and 70 (34.8%) required intensive care unit (ICU) admission; oxygen therapy was required by 117 participants (58.2%) and mechanical ventilation by 79 (39.3%). The mean time from acute COVID-19 infection to assessment was 214.5 ± 116.4 days. All participants had received at least the first dose of a COVID-19 vaccine, as required by the inclusion criteria.

Table 2 presents baseline characteristics stratified by the presence of anosmia and ageusia. Female participants had a higher prevalence of anosmia (*p* = 0.003). Participants with anosmia had significantly lower skeletal muscle mass than those without (28.1 ± 6.6 vs. 31.1 ± 8.1 kg; *p* = 0.004). Significant differences were observed across acute infection severity categories (*p* = 0.003 for anosmia; *p* = 0.004 for ageusia), with a lower prevalence of anosmia and ageusia among participants admitted to the ICU (25.0% vs. 47.2% and 25.6% vs. 48.8%, respectively). Among hospitalized participants, those requiring mechanical ventilation had a lower prevalence of anosmia (44.1% vs. 72.1%; *p* = 0.002) and ageusia (47.4% vs. 71.7%; *p* = 0.005). No other significant differences were observed between groups.

**Table 2:** Baseline stratification by groups

	Anosmia (*n* = 112)	No anosmia (*n* = 89)		Ageusia (*n* = 121)	No ageusia (*n* = 80)	
Age	46.3 ± 12.9	49.5 ± 12.5	p = 0.079	46.4 ± 12.9	49.7 ± 12.5	p = 0.072
Gender						
Male	48 (45.7%)	57 (54.3%)	p = 0.003	57 (54.3%)	48 (45.7%)	p = 0.084
Female*	64 (66.7%)	32 (33.3%)		64 (66.7%)	32 (33.3%)	
Medical history						
SAS	38 (33.9%)	26 (29.2%)	p = 0.543	42 (34.7%)	22 (27.5%)	p = 0.354
Diabetes Mellitus	18 (16.1%)	14 (15.7%)	p = 1.000	19 (15.7%)	13 (16.3%)	p = 1.000
Dyslipidemia	19 (17.0%)	18 (20.2%)	p = 0.586	20 (16.5%)	17 (21.3%)	p = 0.458
Smoking^⸶^	23 (20.5%)	17 (19.1%)	p = 0.860	24 (19.8%)	16 (20.0%)	p = 1.000
Anthropometry and body composition						
BMI (kg/m²)	31.0 ± 6.0	31.0 ± 5.5	p = 0.938	31.3 ± 5.9	30.6 ± 5.5	p = 0.407
SMM (kg)*	28.1 ± 6.6	31.1 ± 8.1	p = 0.004	28.7 ± 7.0	30.5 ± 8.1	p = 0.098
FM (kg)	32.1 ± 12.9	33.6 ± 12.4	p = 0.414	32.7 ± 12.7	32.9 ± 12.7	p = 0.946
BF (%)	38.1 ± 10.7	37.1 ± 9.2	p = 0.460	38.1 ± 10.3	36.9 ± 9.7	p = 0.392
Acute disease severity						
NH*	44 (35.7%)	21 (23.6%)	p = 0.003	45 (37.2%)	20 (25.0%)	p = 0.004
Ward	40 (39.3%)	26 (29.2%)		45 (37.2%)	21 (26.3%)	
ICU	28 (25.0%)	42 (47.2%)		31 (25.6%)	39 (48.8%)	
Infection-evaluation time (d)	221.4 ± 129.0	205.7 ± 98.3	p = 0.344	214.9 ± 124.1	213.8 ± 104.4	p = 0.945
Mechanical ventilation*	30 (44.1%)	49 (72.1%)	p= 0.002	36 (47.4%)	43 (71.7%)	p = 0.005

Categorical data as *n* (%); continuous as mean ± standard deviation; BF = body fat percentage; BMI = body mass index; FM = fat mass; ICU = intensive care unit; NH = non hospitalized; SAS = systemic arterial hypertension; SMM = skeletal muscle mass; ⸶ = currently or former smoker; ‡ Among hospitalized participants; * = reports a significant difference (p<0.05); categorical data as n (%); continuous as mean ± SD

Table 3 presents the comparison of VO₂peak according to the presence of anosmia and ageusia. No significant differences were found between VO₂peak and anosmia (24.1 ± 7.0 vs. 23.0 ± 8.9 mL/kg/min; mean difference 1.13; Cohen’s d = 0.14, 95% CI − 0.14 to 0.42; *p* = 0.325) or ageusia (24.3 ± 7.3 vs. 22.9 ± 8.5 mL/kg/min; mean difference 1.36; Cohen’s d = 0.17, 95% CI − 0.12 to 0.45; *p* = 0.244). A significant difference was found across duration-of-anosmia categories (*p* = 0.038), with higher VO₂peak values in the 2–4 months group than in the < 2 months group; however, this difference did not remain significant after Bonferroni correction (*p* = 0.228). No significant difference was found across duration-of-ageusia categories (*p* = 0.346).

**Table 3:** Comparison between VO_2_peak and the presence of anosmia/ageusia

	VO_2_peak(mL/kg/min)	*p*-value
No anosmia (n = 89)	24.1 ± 7.0	0.325
Anosmia (n = 112)	23.0 ± 8.9	
< 2 months (n = 79)	22.2 ± 8.4	0.038*
2-4 months (n = 16)	27.3 ± 1.0	
4-6 months (n = 3)	30.6 ± 11.5	
> 6 months (n = 14)	20.6 ± .3	
No ageusia (n = 80)	24.3 ± 7.3	0.244
Ageusia (n = 121)	22.9 ± 8.5	
< 2 months (n = 93)	23.3 ± 8.6	0.346
2-4 months (n = 11)	24.4 ± 11.8	
4-6 months (n = 5)	19.4 ± 7.2	
> 6 months (n = 12)	20.0 ± 4.5	

Data are presented as mean and standard deviation (±); * = reports a significant difference (p<0.05)

Table 4 presents the logistic regression models for the association between anosmia and neurological symptoms. In unadjusted analyses, anosmia was significantly associated with memory deficit (OR = 2.04; 95% CI 1.12–3.72), concentration deficit (OR = 2.00; 95% CI 1.12–3.56), dizziness (OR = 2.47; 95% CI 1.37–4.47), headache (OR = 1.89; 95% CI 1.06–3.38), and aural fullness (OR = 4.78; 95% CI 1.35–16.96). After adjustment for age, sex, acute infection severity, medical history, body fat percentage, and skeletal muscle mass, anosmia remained independently associated with dizziness (OR = 2.22; 95% CI 1.15–4.31) and aural fullness (OR = 4.54; 95% CI 1.16–17.80). No significant associations were found between anosmia and tinnitus (adjusted OR = 1.65; 95% CI 0.73–3.76) or hearing loss (adjusted OR = 1.37; 95% CI 0.50–3.74).

**Table 4:** Logistic regression models comparing anosmia and neurological symptoms.

	Anosmia (*n* = 112)	No anosmia (n = 89)	Unadjusted OR (95%CI)	Adjusted OR (95%CI)*
Memory deficit	84 (75.0)	53 (59.6)	2.04 (1.12 to 3.72)	1.95 (0.99 to 3.84)
Concentration deficit	55 (49.1)	29 (32.6)	2.00 (1.12 to 3.56)	1.59 (0.84 to 3.02)
Dizziness	55 (49.1)	25 (28.1)	2.47 (1.37 to 4.47)	2.22 (1.15 to 4.31)
Tinnitus	29 (25.9)	15 (16.9)	1.72 (0.86 to 3.46)	1.65 (0.73 to 3.76)
Headache	52 (46.4)	28 (31.5)	1.89 (1.06 to 3.38)	1.60 (0.83 to 3.08)
Hearing loss	16 (14.3)	9 (10.1)	1.48 (0.62 to 3.53)	1.37 (0.50 to 3.74)
Aural fullness	16 (14.3)	3 (3.4)	4.78 (1.35 to 16.96)	4.54 (1.16 to 17.80)

Categorical data are presented as absolute and relative frequencies (%). CI = confidence interval; OR = odds ratio; * = models adjusted for: age, gender, acute disease severity, medical history, body fat percentage and skeletal muscle mass; † Adjusted for age, sex, acute infection severity, medical history (arterial hypertension, diabetes mellitus, dyslipidemia, smoking), body fat percentage, and skeletal muscle mass

Table 5 presents the logistic regression models for the association between ageusia and neurological symptoms. In unadjusted analyses, ageusia was significantly associated with memory deficit (OR = 2.72; 95% CI 1.48–5.00), concentration deficit (OR = 2.09; 95% CI 1.16–3.79), dizziness (OR = 1.83; 95% CI 1.01–3.32), headache (OR = 1.83; 95% CI 1.01–3.32), and aural fullness (OR = 3.91; 95% CI 1.10–13.90). After adjustment, ageusia remained independently associated with memory deficit (OR = 2.91; 95% CI 1.47–5.75) and aural fullness (OR = 4.08; 95% CI 1.02–16.30). No significant associations were found between ageusia and tinnitus (adjusted OR = 1.24; 95% CI 0.56–2.75) or hearing loss (adjusted OR = 1.42; 95% CI 0.53–3.82).

**Table 5:** Logistic regression models comparing ageusia and neurological symptoms

	Ageusia (*n* = 121)	No ageusia (*n* = 80)	Unadjusted OR (95%CI)	Adjusted OR (95%CI)*
Memory deficit	93 (76.9)	44 (55.0)	2.72 (1.48 to 5.00)	2.91 (1.47 to 5.75)
Concentration deficit	59 (48.8)	25 (31.3)	2.09 (1.16 to 3.79)	1.81 (0.95 to 3.45)
Dizziness	55 (45.5)	25 (31.3)	1.83 (1.01 to 3.32)	1.79 (0.93 to 3.46)
Tinnitus	28 (25.0)	16 (20.0)	1.20 (0.60 to 2.41)	1.24 (0.56 to 2.75)
Headache	55 (45.5)	25 (31.3)	1.83 (1.01 to 3.32)	1.72 (0.88 to 3.37)
Hearing loss	17 (14.0)	8 (10.0)	1.47 (0.60 to 3.59)	1.42 (0.53 to 3.82)
Aural fullness	16 (13.2)	3 (3.8)	3.91 (1.10 to 13.90)	4.08 (1.02 to 16.30)

Categorical data are presented as absolute and relative frequencies (%). CI = confidence interval; OR = odds ratio; * = Models adjusted for: age, gender, acute disease severity, medical history, body fat percentage and skeletal muscle mass; † Adjusted for age, sex, acute infection severity, medical history (arterial hypertension, diabetes mellitus, dyslipidemia, smoking), body fat percentage, and skeletal muscle mass

## Discussion

The results of this study involving post-COVID-19 individuals indicated that anosmia and ageusia were associated with neurological symptoms. After adjustment for potential confounders, anosmia was independently associated with dizziness and aural fullness, whereas ageusia was independently associated with memory deficit and aural fullness. No significant associations were found between anosmia or ageusia and tinnitus or hearing loss. Animal studies investigating severe acute respiratory syndrome coronavirus 2 (SARS-CoV-2) have implicated the angiotensin-converting enzyme 2 receptor as a mediator of coronavirus-related neuronal damage and have shown that SARS-CoV-2 may infect the cerebrovascular endothelium predominantly, as well as the brain parenchyma, particularly in the medial temporal lobe, resulting in apoptosis and necrosis. Postmortem investigations of human brains indicate that human coronavirus variants and SARS-CoV-2 may infect neurons and glial cells, suggesting that SARS-CoV-2 may exhibit similar neurovirulence. In addition, analyses have demonstrated increased serum cytokine levels following SARS-CoV infection, supporting the hypothesis that cytokine overproduction and toxicity may represent a potential mechanism of neurological injury, paralleling a known pathway of pulmonary damage [14].

Early reports suggest that COVID-19 may be associated with severe neurological complications, and several plausible mechanisms may explain these observations. Greater awareness of the potential neurological involvement of the disease, along with further investigation into the underlying pathophysiology, is necessary to understand and ultimately mitigate SARS-CoV-2–associated neurological injury. This may help explain the high prevalence of neurological symptoms in our study population, which consisted predominantly of individuals with comorbidities such as hypertension, diabetes, obesity, and advanced age, conditions that may increase the risk of immune dysregulation [14–16]. Furthermore, the findings of the present study are consistent with research aimed at determining whether neurological symptoms are associated with COVID-19 and at characterizing headache manifestations. In that study, 288 patients presenting at least one neurological symptom were interviewed, and 22.2% reported that one of these symptoms was the most bothersome during COVID-19.

The neurological symptoms reported were ageusia (69.8%), headache (69.1%), anosmia (67.0%), myalgia (44.4%), drowsiness (37.2%), agitation (20.8%), mental confusion (14.9%), syncope (4.9%), and seizures (2.8%). Among women, those who experienced fever, sore throat, anosmia/ageusia, and myalgia also reported significantly more headache. Headache is frequent in COVID-19, is associated with other symptoms such as fever, sore throat, anosmia, ageusia, and myalgia, and may persist beyond the acute phase of the disease [16]. The findings of this study regarding the association of anosmia and ageusia with neurological symptoms, including memory impairment, dizziness, and aural fullness, together with the absence of an association with VO₂peak, are consistent with the literature suggesting that post-COVID olfactory dysfunction may serve as a marker of neurocognitive impairment and neuropsychiatric symptoms. Some studies have identified frequent associations between olfactory loss and poorer cognitive performance, in addition to reports of “brain fog” and headache in patients with altered olfaction after SARS-CoV-2 infection [17, 18].

Morphological and molecular evidence indicates a process involving central neuroinflammation that may explain this clinical association. Such mechanisms include alterations in perfusion and connectivity in frontal and limbic regions among patients with persistent anosmia, as well as increased inflammatory and neuropeptide markers in the olfactory pathway, such as overexpression of Substance P and Prokineticin-2 in olfactory neurons. These findings suggest a local neuroinflammatory mechanism with possible propagation to brain networks responsible for attention and executive control [19, 20]. This evidence strengthens the biological plausibility that sensory alterations involving both smell and taste may correlate with neurological symptoms, likely reflecting involvement of the central nervous system and persistent dysfunction of the olfactory pathway, even in the absence of cardiorespiratory impairment measured by VO₂peak.

Conversely, objective measures of cardiorespiratory fitness based on peak VO₂ have yielded heterogeneous findings in post-COVID-19 populations. Some studies have reported reductions in mean VO₂ among patients with persistent symptoms. However, these reductions appear to be more strongly associated with a history of severe disease, dyspnea, physical deconditioning, autonomic dysfunction, or peripheral limitations than with olfactory sensory dysfunction itself [21, 22]. Therefore, the absence of an association between anosmia or ageusia and VO₂peak in the present study is compatible with the hypothesis that anosmia primarily reflects a central neuroinflammatory phenotype of post-acute sequelae of SARS-CoV-2 infection, whereas reduced VO₂peak reflects cardiopulmonary, muscular, or autonomic mechanisms that do not necessarily coexist with sensory alterations. Indeed, the observed differences in VO₂peak between groups were small and non-significant (mean difference 1.13 mL/kg/min, Cohen’s d = 0.14, 95% CI − 0.14 to 0.42, *p* = 0.325, for anosmia; mean difference 1.36 mL/kg/min, Cohen’s d = 0.17, 95% CI − 0.12 to 0.45, *p* = 0.244, for ageusia), supporting the interpretation that neurological manifestations and exercise capacity represent partially independent post-COVID phenotypes.

Based on the findings of the present study, several practical implications and future research directions may be proposed. Future studies on post-acute sequelae of SARS-CoV-2 infection should consider stratifying clinical phenotypes, for example, distinguishing a neurological phenotype characterized by anosmia and brain fog from a cardiorespiratory phenotype characterized by exercise intolerance. Although a nominal difference in VO₂peak was observed across duration-of-anosmia categories, it did not remain significant after Bonferroni correction, indicating that symptom duration did not meaningfully influence cardiorespiratory fitness. In addition, multidisciplinary assessments, including neuropsychological testing, olfactometry, functional neuroimaging, and cardiopulmonary exercise testing, may help clarify underlying mechanisms and guide targeted interventions [17, 22].

Although this topic remains relatively underexplored, it is highly relevant for post-COVID-19 populations, particularly because neurological and functional symptoms form the basis of many evaluations related to Long COVID. Health professionals involved in the care of post-COVID-19 individuals require adequate knowledge and strategies to avoid compromising the quality of treatment. Several limitations of the present study should be acknowledged. First, because the questionnaire relied on subjective self-reporting of neurological symptoms, and of anosmia and ageusia, which were not assessed by objective olfactometry or gustometry, the possibility of recall bias and misclassification should be considered. Second, the cross-sectional design precludes causal inference and does not allow the establishment of cause-and-effect relationships between anosmia or ageusia and neurological symptoms. Third, the absence of objective neuropsychological testing limits the interpretation of the memory and concentration deficits reported by participants. Fourth, the lack of longitudinal follow-up prevents evaluation of symptom evolution over time. Fifth, the study population was recruited from hospital-discharge referrals, with 67.7% of participants hospitalized and 34.8% requiring ICU admission during the acute infection; the findings therefore reflect a predominantly severe clinical spectrum and may not be generalizable to the full post-COVID population. Sixth, baseline physical activity level was not systematically measured and may influence VO₂peak independently of anosmia or ageusia.

Finally, although all associations remained significant after Benjamini–Hochberg correction for multiple comparisons, several were borderline and should be interpreted with caution. Nevertheless, this study provides relevant and novel findings regarding the relationship between anosmia and ageusia and neurological symptoms among post-COVID-19 individuals, as well as the cardiorespiratory fitness of patients presenting these sensory symptoms. Understanding these associations may contribute to the development of health promotion and care interventions in the post-pandemic period, potentially improving overall health and quality of life among individuals with Long COVID. Studies addressing the neurological impact of COVID-19 may help inform clinical management and underscore the importance of further investigation into Long COVID.

## Conclusion

In conclusion, anosmia and ageusia were independently associated with neurological symptoms in post-COVID-19 individuals, with anosmia associated with dizziness and aural fullness and ageusia associated with memory deficit and aural fullness, whereas no significant association was observed between these sensory symptoms and cardiorespiratory fitness. These findings support the interpretation that neurological and cardiorespiratory manifestations may represent partially independent post-COVID phenotypes. From a clinical perspective, the presence of anosmia and ageusia may serve as readily accessible markers to identify individuals at greater risk of cognitive or neurological sequelae, supporting the early integration of targeted neuropsychological assessment and structured follow-up into rehabilitation planning. Nonetheless, the cross-sectional design and the self-reported nature of the symptoms preclude causal inferences, and longitudinal studies incorporating objective olfactory, gustatory, and neuropsychological assessments are warranted to confirm these associations. Given the burden of Long COVID, the history of anosmia and ageusia should be systematically considered in the therapeutic assessment and management of patients with persistent cognitive symptoms.

## Data Availability

The data that support the findings of this study are available from the corresponding author upon reasonable request.

## References

[1] Suzuki M, Saito K, Min WP, Vladau C, Toida K, Itoh H (2007) Identification of viruses in patients with postviral olfactory dysfunction. Laryngoscope 117(2):272–277. 10.1097/01.mlg.0000249922.37381.1e PMC7165544 17277621

[2] Costa Klinger VT, Carnaúba ATL, Rocha KW, Andrade KCL, Ferreira SMS, Menezes PL (2020) Olfactory and taste disorders in COVID-19: a systematic review. Braz J Otorhinolaryngol 86(6):781–792. 10.1016/j.bjorl.2020.05.008 PMC7280089 32580925

[3] Allis TJ, Leopold DA (2012) Smell and taste disorders. Facial Plast Surg Clin North Am 20(1):93–111. 10.1016/j.fsc.2011.10.011 22099622

[4] Novak P, Mukerji SS, Alabsi HS, Systrom D, Marciano SP, Felsenstein D, Mullally WJ, Pilgrim DM (2022) Multisystem Involvement in Post-Acute Sequelae of Coronavirus Disease 19. Ann Neurol 91(3):367–379. 10.1002/ana.26286 PMC9011495 34952975

[5] Afrisham R, Jadidi Y, Davoudi M, Moayedi K, Karami S, Sadegh-Nejadi S, Ashtary-Larky D, Seyyedebrahimi S, Alizadeh S (2022) Renal, cardiac, neurological, cutaneous and coagulopathic long-term manifestations of COVID-19 after recovery; A review. Epidemiol Infect 150:e208. 10.1017/S0950268822001480 PMC10212601 36128671

[6] Marchiori LL, de Ciquinato M, de Marchiori DS, de Silva G, Okamura LHK, Branco LST (2023) BHM. Estudo comparativo de zumbido e aptidão cardiorrespiratória entre pessoas pós-COVID-19. Audiol, Commun Res [Internet]. ;28:e2774. 10.1590/2317-6431-2023-2774pt

[7] Marchiori G, de Branco M, Ciquinato BHM, de Gehren D, Carvalho AD, Mesti GH (2022) Anosmia and ageusia in people after COVID-19: an analysis between the type and length of hospital stay. Rev CEFAC [Internet] 24(2):e9322. 10.1590/1982-0216/20222429322

[8] Elvan-Tuz A, Karadag-Oncel E, Kiran S, Kanik-Yuksek S, Gulhan B, Hacimustafaoglu M, Ozdem-Alatas S, Kuyucu N, Ozdemir H, Egil O, Elmas-Bozdemir S, Polat M, Bursal-Duramaz B, Cem E, Apaydin G, Teksam O, TURK-COVID-19-Anosmia Study Group (2022) Prevalence of Anosmia in 10.157 Pediatric COVID-19 Cases: Multicenter Study from Turkey. Pediatr Infect Dis J 41(6):473–477. 10.1097/INF.0000000000003526 PMC9083317 35349499

[9] Jung YH, Ha EH, Park J, Choe KW, Lee WJ, Jo DH (2023) Neurological and Psychiatric Manifestations of Post-COVID-19 Conditions. J Korean Med Sci 38(11):e83. 10.3346/jkms.2023.38.e83 PMC10027539 36942392

[10] Arango-Ibanez JP, Córdoba-Melo BD, Gutiérrez Posso JM, Barbosa-Rengifo MM, Herrera CJ, Quintana Da Silva MA, Buitrago AF, Coronel Gilio ML, Pow-Chong-Long F, Gómez-Mesa JE (2024) Long COVID Clusters of Symptoms Persist beyond Two Years after Infection: Insights from the CARDIO COVID 20–21 Registry. Viruses 16(7):1028. 10.3390/v16071028 PMC11281355 39066191

[11] Chiaramonte R, Civello T, Laganga Senzio G, Longo L, De Caro AS, Li Gotti F, Vecchio M (2025) Multidimensional stratification of severe disability: demographic, clinical, geographic, socio-economic profiles and healthcare pathways in a cross-sectional Italian cohort. Healthcare 13(24):3200. 10.3390/healthcare13243200 PMC12732793 41464269

[12] von Elm E, Altman DG, Egger M, Pocock SJ, Gøtzsche PC, Vandenbroucke JP (2007) The Strengthening the Reporting of Observational Studies in Epidemiology (STROBE) Statement: guidelines for reporting observational studies. Ann Intern Med 147(8):573–577 10.7326/0003-4819-147-8-200710160-00010 17938396

[13] Lemos MM, Cavalini GR, Pugliese Henrique CR, Perli VAS, de Moraes Marchiori G, Marchiori LLM, Sordi AF, Franzói de Moraes SM, de Paula Ramos S, Valdés-Badilla P, Mota J, Magnani Branco BH (2022) Body composition and cardiorespiratory fitness in overweight or obese people post COVID-19: A comparative study. Front Physiol 13:949351. 10.3389/fphys.2022.949351 PMC9532828 36213222

[14] Aghagoli G, Gallo Marin B, Katchur NJ, Chaves-Sell F, Asaad WF, Murphy SA (2021) Neurological Involvement in COVID-19 and Potential Mechanisms: A Review. Neurocrit Care 34(3):1062–1071. 10.1007/s12028-020-01049-4 PMC7358290 32661794

[15] Piotrowicz K, Gasowski J, Michel JP, Veronese N (2021) Post-COVID-19 acute sarcopenia: physiopathology and management. Aging Clin Exp Res 33(10):2887–2898. 10.1007/s40520-021-01942-8 PMC8323089 34328636

[16] Sampaio Rocha-Filho PA, Albuquerque PM, Carvalho LCLS, Dandara Pereira Gama M, Magalães JE (2022) Headache, anosmia, ageusia and other neurological symptoms in COVID-19: a cross-sectional study. J Headache Pain 23(1):2. 10.1186/s10194-021-01367-8 PMC8721484 34979899

[17] Vilarello BJ, Jacobson PT, Tervo JP, Waring NA, Gudis DA, Goldberg TE, Devanand DP, Overdevest JB (2023) Olfaction and neurocognition after COVID-19: a scoping review. Front Neurosci 17:1198267. 10.3389/fnins.2023.1198267 PMC10339825 37457004

[18] Di Stadio A, Brenner MJ, De Luca P, Albanese M, D’Ascanio L, Ralli M, Roccamatisi D, Cingolani C, Vitelli F, Camaioni A, Di Girolamo S, Bernitsas E (2022) Olfactory Dysfunction, Headache, and Mental Clouding in Adults with Long-COVID-19: What Is the Link between Cognition and Olfaction? A Cross-Sectional Study. Brain Sci 12(2):154. 10.3390/brainsci12020154 PMC8870047 35203918

[19] Schirinzi T, Lattanzi R, Maftei D, Grillo P, Zenuni H, Boffa L, Albanese M, Simonetta C, Bovenzi R, Maurizi R, Loccisano L, Vincenzi M, Greco A, Di Girolamo S, Mercuri NB, Passali FM, Severini C (2023) Substance P and Prokineticin-2 are overexpressed in olfactory neurons and play differential roles in persons with persistent post-COVID-19 olfactory dysfunction. Brain Behav Immun 108:302–308. 10.1016/j.bbi.2022.12.017 PMC9760596 36549578

[20] Yus M, Matias-Guiu JA, Gil-Martínez L, Gómez-Ruiz N, Polidura C, Jorquera M, Delgado-Alonso C, Díez-Cirarda M, Matías-Guiu J, Arrazola J (2022) Persistent olfactory dysfunction after COVID-19 is associated with reduced perfusion in the frontal lobe. Acta Neurol Scand 146(2):194–198. 10.1111/ane.13627 PMC9111206 35467007

[21] Durstenfeld MS, Sun K, Tahir P, Peluso MJ, Deeks SG, Aras MA, Grandis DJ, Long CS, Beatty A, Hsue PY (2022) Use of Cardiopulmonary Exercise Testing to Evaluate Long COVID-19 Symptoms in Adults: A Systematic Review and Meta-analysis. JAMA Netw Open 5(10):e2236057. 10.1001/jamanetworkopen.2022.36057 PMC9557896 36223120

[22] Schwendinger F, Knaier R, Radtke T, Schmidt-Trucksäss A (2023) Low Cardiorespiratory Fitness Post-COVID-19: A Narrative Review. Sports Med 53(1):51–74. 10.1007/s40279-022-01751-7 PMC9483283 36115933

